# Cisplatin palbociclib combination differentially modulates PTEN AKT signaling via Hsp90 in hepatocellular carcinoma cells

**DOI:** 10.1038/s41598-025-04008-1

**Published:** 2025-06-02

**Authors:** Hameis M. Sleem, Aya A. Ali, Eman Ramadan

**Affiliations:** 1 Department of Pharmacology and Biochemistry, Faculty of Pharmacy, The British University in Egypt, El Sherouk City, Egypt; 2 Drug Research and Development Group (DRD), Health Research Center of Excellence, The British University in Egypt, El Sherouk City, Egypt

**Keywords:** Cisplatin, Palbociclib, HCC, PTEN, Hsp90, AKT, Biological techniques, Cancer, Genetics, Diseases

## Abstract

Hepatocellular carcinoma (HCC) presents a significant global health challenge, marked by high mortality and recurrence. This study investigated the synergistic potential of cisplatin and palbociclib (C + P) against HCC cell lines. RT-qPCR revealed that C + P significantly downregulated HCC-related genes, including Hsp90, β-catenin, and components of the PI3K/AKT/mTOR pathway, compared to cisplatin alone and controls. Western blotting confirmed a reduction in phosphorylated AKT (P-AKT) with palbociclib and C + P, while PTEN, a tumor suppressor, was upregulated in the C + P group. Annexin V-FITC assays demonstrated a substantial increase in apoptosis in palbociclib and C + P treated cells. Cell cycle analysis indicated S and G0–G1 phase arrest with C + P, suggesting a combined cytotoxic effect. Scratch wound assays showed that both palbociclib and C + P significantly inhibited cell migration compared to cisplatin and controls. These findings suggest a promising synergistic effect of C + P in overcoming cisplatin resistance in HCC. However, further research is needed to fully elucidate the complex interactions between these drugs.

**Supplementary Information:**

The online version contains supplementary material available at 10.1038/s41598-025-04008-1.

## Introduction

Hepatocellular carcinoma (HCC), originating from hepatocytes, constitutes the predominant form of primary hepatic malignancy, posing a significant global public health burden. Despite advancements in diagnosis and treatment strategies, HCC remains associated with a high mortality rate among cancer patients^1^. The optimal treatment approach for HCC depends on various factors, including tumor stage, underlying liver function, and patient health status. Nevertheless, contemporary therapeutic interventions, encompassing surgical resection, liver transplantation, and locoregional modalities, frequently exhibit constraints^2^.

Cisplatin (CDDP), a platinum-based chemotherapeutic drug, has been used for decades in HCC treatment regimens. Its effectiveness lies in its ability to disrupt DNA replication and induce cell death in cancer cells. Cisplatin, a broad-spectrum cell cycle inhibitor, exerts its antitumor effect on HCC through its cytotoxic properties. This platinum-based chemotherapeutic agent primarily targets nuclear macromolecules, including DNA and RNA, by forming DNA adducts and intra-strand cross-links. Additionally, cisplatin can interact with nuclear and cytoplasmic proteins, further contributing to its DNA-damaging activity. Consequently, cisplatin is classified as a DNA-alkylating agent^3–5^. However, the usefulness of cisplatin is significantly compromised by the development of cisplatin resistance, a major obstacle in HCC therapy^6^. This resistance arises from various mechanisms employed by cancer cells, including increased drug efflux, enhanced DNA repair pathways, and alterations in cellular signaling cascades that promote cell survival despite DNA damage^7^. When cisplatin resistance emerges, treatment efficacy decreases, leading to tumor recurrence, poorer prognosis, and a desperate need for novel therapeutic strategies.

The retinoblastoma (Rb) pathway functions as an essential regulatory mechanism, governing the G1 to S phase cell cycle transition. This pathway’s operation is contingent upon cyclin-dependent kinase (CDK)4/6 and cyclin D mediated phosphorylation of the Rb protein, resulting in Rb inactivation. Subsequently, the E2F transcription factor is released, initiating the progression of the cell cycle^8–10^. Recent studies had unveiled broader functions for CDK4/6, cyclin D, and E2F. These molecules appear to be involved in various cellular processes, including DNA repair, programmed cell death, cellular differentiation, and regulation of the immune system^11^. Perturbation of the retinoblastoma (Rb) pathway, a key modulator of cell cycle advancement, has prompted the evolution of CDK4/6 inhibitors as a treatment strategy for breast carcinoma. Palbociclib, ribociclib, and abemaciclib constitute a group of CDK4/6 inhibitors that have received clinical authorization to impede this pathway within breast neoplasms exhibiting Rb pathway aberrations^12,13^. Similar pathway aberrations are frequently observed in hepatocellular carcinoma (HCC), suggesting potential therapeutic applications for CDK4/6 inhibitors in this context as well^14–17^.

Palbociclib, a selective inhibitor of cyclin-dependent kinases 4/6 (CDK4/6), has demonstrated therapeutic potential across diverse malignancies. CDK4/6, in complex with cyclin D, modulates cell cycle progression by phosphorylating the retinoblastoma protein (Rb), facilitating the G1 to S phase transition and promoting neoplastic cell proliferation^18^. Palbociclib abrogates this mechanism by specifically suppressing CDK4/6 activity, resulting in cell cycle arrest and impeding tumor cell growth^19^.

The potential for combining palbociclib and cisplatin in HCC therapy is particularly exciting for several reasons. Firstly, this combination strategy targets distinct cellular pathways. Cisplatin directly damages DNA, while palbociclib disrupts cell cycle progression. This multi-pronged approach could potentially overcome resistance mechanisms developed against cisplatin alone. Secondly, there’s a possibility of synergy between the two drugs. Palbociclib might enhance the effectiveness of cisplatin by sensitizing cancer cells to DNA damage or by impeding their ability to repair cisplatin-induced DNA lesions. This synergistic effect could lead to a more potent anti-cancer effect compared to either drug used individually^20^. Beyond their distinct mechanisms of action, the combination of palbociclib and cisplatin might hold promise in HCC therapy by targeting key signaling pathways involved in HCC development and progression.

The PI3K/AKT/mTOR signaling cascade acts as a master regulator of cellular processes critical for growth, proliferation, and survival. Aberrant activation of this pathway is frequently observed in HCC. Palbociclib, by inhibiting CDK4/6, can indirectly suppress the PI3K/AKT/mTOR pathway by hindering cell cycle progression downstream. Cisplatin might also contribute to pathway inhibition by inducing DNA damage, leading to feedback mechanisms that reduce PI3K/AKT/mTOR signaling^21,22^.

The Wnt/β-catenin pathway is another critical regulator of cell proliferation and differentiation. In the context of HCC, dysregulation of this pathway can promote tumor growth. Palbociclib’s effect on cell cycle arrest might indirectly impact the Wnt/β-catenin pathway by limiting β-catenin accumulation and its role in downstream gene expression. Cisplatin, on the other hand, might have less direct effects on this pathway, but its DNA damaging properties could still influence its activity through complex cellular responses^23,24^.

Heat shock protein 90 (HSP90) functions as a molecular chaperone, facilitating the proper folding and stabilization of a diverse array of oncogenic proteins. By enhancing the function of these cancer-promoting proteins, HSP90 contributes to the process of tumorigenesis. Targeting HSP90 can lead to the degradation of these client proteins. While palbociclib doesn’t directly target HSP90, cisplatin can induce HSP90 dysfunction, leading to the degradation of several oncogenic client proteins, potentially contributing to its anti-tumor effects^25,26^.

Investigating the precise effects of the palbociclib and cisplatin combination on these pathways in HCC cells is crucial for understanding the mechanisms underlying their potential synergy and improved therapeutic efficacy. This knowledge can pave the way for optimizing treatment strategies and potentially overcoming cisplatin resistance in HCC patients.

In light of these promising prospects, this study presents a novel approach to addressing cisplatin resistance in hepatocellular carcinoma (HCC). While the combination of palbociclib and cisplatin has been explored in other cancer types, this study represents a pioneering application in HCC, given the unique biological characteristics of this disease. By combining these two drugs, we aim to explore the underlying mechanisms by which this combination therapy exerts its anti-cancer effects and evaluate its efficacy in enhancing cytotoxicity in HCC experimental cell models, specifically HepG2 and Huh-7. This study offers novel insights into the mechanisms of action that are crucial for cancer cell survival and growth.

## Experimental procedures

### Materials

The study utilized two human hepatocellular carcinoma (HCC) cell lines, HepG2 (TCHu72) and Huh-7 (TCHu182), sourced from VACSERA (Giza, Egypt). Pharmaceutical-grade cisplatin and palbociclib were obtained from a local pharmacy (Cairo, Egypt). Cell culture was performed using Dulbecco’s Modified Eagle Medium (DMEM) supplemented with fetal bovine serum (FBS), both procured from Gibco® (Thermo Fisher Scientific, USA). An antibiotic–antimycotic solution, containing penicillin (100 U/mL), streptomycin (0.1 U/mL), and amphotericin B (0.25 μg/mL), was acquired from Lonza® (Walkersville, Maryland, USA). All remaining chemical reagents, unless otherwise stated, were purchased from Sigma Aldrich (USA).

### Cell culture

For cellular propagation, Huh-7 and HepG2 lines were sustained in a supplemented Dulbecco’s Modified Eagle Medium (DMEM), enriched with 10% fetal bovine serum (FBS), penicillin (100 U/mL), streptomycin (0.1 U/mL), and amphotericin B (0.25 μg/mL). A humidified environment, maintained at 37 °C with 5% CO_2_, was utilized for incubation. All cell culture manipulations were performed under aseptic conditions within a Class II laminar flow hood. Temperature was held at 37 °C for all incubations, unless otherwise noted.

### Cell viability assays

To determine cellular viability, an MTT assay, utilizing the Invitrogen Vybrant® MTT Cell Proliferation Assay Kit, was performed. Cells were seeded into 96-well plates at a density of 10^4^ cells per well and allowed to incubate overnight. Following this, cells were exposed to increasing concentrations of cisplatin (0.3–10 µM) and palbociclib (0.3–5 µM) for a 48-h period. After treatment, the medium was exchanged for DMEM containing MTT at a final concentration of 0.5 mg/mL, and the cells were incubated for an additional 2 h at 37 °C. Subsequently, the medium was aspirated, and the formed formazan crystals were dissolved in DMSO. Absorbance was then measured at 570 nm. The IC50 values were calculated using GraphPad Prism software, employing nonlinear regression analysis.

### Combination analysis

To evaluate the combined impact of cisplatin and palbociclib, cells were seeded according to the aforementioned protocol. Following an overnight adherence period, cells were treated for 48 h with escalating concentrations of palbociclib (0.3–5 µM), both independently and in conjunction with a constant cisplatin concentration (IC30) which is 1.83 µM in HepG2 and 2.08 µM in Huh-7. The interaction between the two drugs was quantified using the combination index (CI), derived from Chou’s^27^ isobologram equation. This approach compares the individual drug concentrations (Dn) needed to achieve a specific effect (n%) against those required when used in combination (D). The CI was determined using the following formula:

$${\text{CI}} = \left( {{\text{D}}_{1} } \right)/\left( {{\text{D}}_{{\text{n}}} } \right)_{{1}} + \left( {{\text{D}}_{2} } \right)/\left( {{\text{D}}_{{\text{n}}} } \right)_{2}$$

In this equation, (D_n_)_1_ and (D_n_)_2_ represent the concentrations of each individual drug required to achieve an n% effect, while (D_1_) and (D_2_) denote the concentrations of these drugs when combined to produce the same effect. A combination index (CI) below 0.8 signifies synergism, indicating that the combined treatment of cisplatin and palbociclib yields a greater effect than the sum of their individual effects at the applied concentrations. A CI ranging from 0.8 to 1.264 suggests additivity, where the combination’s effect is consistent with the sum of the individual drug effects. Conversely, a CI above 1.2 implies antagonism, meaning the combination’s effect is less than anticipated based on their individual activities.

### RNA extraction and cDNA synthesis

For gene expression analysis, Huh-7 and HepG2 cells were introduced into T-75 flasks at a density of 2 × 10^6^ cells per flask and allowed to attach overnight. The next day, fresh DMEM was used to replace the existing medium. To investigate the effects of cisplatin, palbociclib, and their combination on gene expression, HepG2 and Huh-7 cells were treated under four conditions: untreated control with DMEM medium alone; cisplatin-only medium (1.83 µM for HepG2, 2.08 µM for Huh-7); palbociclib-only medium (1.016 µM for HepG2, 1.336 µM for Huh-7); and combined cisplatin plus palbociclib (1.83 µM + 0.2938 µM for HepG2, 2.08 µM + 0.463 µM for Huh-7). All treatments were applied for 48 h. These concentrations were consistently used in all subsequent experiments. Total RNA was then isolated using the Direct-zol™ RNA MiniPrep Kit (Zymo Research, USA, Cat. No. R2051), in accordance with the manufacturer’s guidelines. The quality and quantity of the isolated RNA were assessed by measuring the A260/A280 ratio using a Q5000 UV–Vis Nanodrop spectrophotometer (Quawell, USA). Subsequently, the extracted RNA was converted to complementary DNA (cDNA) using the GoScript Reverse Transcription Kit (Promega, USA, Cat. No. A5000), as per the manufacturer’s instructions. This process generated stable cDNA, suitable for downstream applications like quantitative PCR.

### Quantitative real-time PCR (qRT-PCR)

Gene expression levels were quantified using quantitative real-time PCR (qRT-PCR). Reactions were carried out in a 20 µL volume using the StepOne Plus Real-Time PCR System (Applied Biosystems, USA). Each well of a 96-well plate was prepared with 10 µL GoTaq® qPCR Master Mix (Promega, USA, Cat No. A6001), 0.4 µM forward and reverse primers, and 2 µL of diluted cDNA from each reverse transcription reaction. Primers were obtained from HS Thermo Fisher Scientific, USA. Relative gene expression was determined using the 2^−ΔΔCT^ ± standard deviation method. Primer sequences are detailed in Table 1.

**Table 1:** List of the primer sequences employed in the qRT-PCR.

Gene	Forward primer	Reverse primer
HSP90	TCTGCCTCTGGTGATGAGATGG	CGTTCCACAAAGGCTGAGTTAGC
B catenin	CACAAGCAGAGTGCTGAAGGTG	GATTCCTGAGAGTCCAAAGACAG
GSK3B	CCGACTAACACCACTGGAAGCT	AGGATGGTAGCCAGAGGTGGAT
PIK3CA	GAAGCACCTGAATAGGCAAGTCG	GAGCATCCATGAAATCTGGTCGC
AKT1	TGGACTACCTGCACTCGGAGAA	GTGCCGCAAAAGGTCTTCATGG
m-TOR	AGCATCGGATGCTTAGGAGTGG	CAGCCAGTCATCTTTGGAGACC
BCL2	AAGATTGATGGGATCGTTGC	GCGGAACACTTGATTCTGGT
Beta actin	CACCATTGGCAATGAGCGGTTC	AGGTCTTTGCGGATGTCCACGT

### Western blot analysis

Protein expression was evaluated through Western blot analysis. Cell samples underwent lysis in RIPA buffer, containing protease and phosphatase inhibitors. Subsequent to homogenization and sonication, lysates were cleared by centrifugation. Protein levels were determined using the BCA method. Equivalent protein amounts (25–50 μg) were resolved by electrophoresis on 6–10% SDS-PAGE gels and then transferred to PVDF membranes. To reduce non-specific binding, membranes were blocked with 5% non-fat milk in TBST. Membranes were then incubated with primary antibodies against PTEN, phosphorylated AKT (P-AKT), and GAPDH, with GAPDH functioning as a normalization control. Following the removal of unbound primary antibodies, membranes were incubated with HRP-conjugated secondary antibodies, specific to the host species of the primary antibodies. Protein bands were visualized using ECL substrate and quantified via densitometry with image analysis software. The primary antibodies utilized were: PTEN (FAGUS, Cat.FAS-81483-A), P-AKT (Invitrogen, Cat.PA5-95669), and GAPDH (Invitrogen, Cat.MA5-15738). The secondary antibodies were HRP-conjugated anti-mouse IgG (Invitrogen) and anti-rabbit IgG (Invitrogen).

### Scratch wound assay

To assess the impact of cisplatin, palbociclib, and their combined application on Huh-7 and HepG2 cell migration and proliferation, an in vitro wound healing assay was performed. Cells were seeded in 6-well plates and grown until they reached 80–90% confluence. A consistent scratch wound was created in each well using a 200 µL pipette tip. Following this, the wells were rinsed twice with PBS to eliminate detached cells. Cells were then exposed to either complete DMEM medium or medium supplemented with cisplatin, palbociclib, or their combination. Wound closure was observed over a 48-h period after treatment. Images were captured at 0, 24, and 48 h using an inverted microscope (Labomed Inc., LA, CA, USA) connected to a digital camera. Wound healing was quantified using the ImageJ® plugin tool (NIH, USA), according to the method described by Suarez-Arnedo et al.^28^. This methodology allowed for a comparative analysis of the treatments’ effects on cellular wound healing, potentially revealing their influence on cell migration and proliferation.

### Annexin V/propidium iodide (PI) apoptosis assay on HepG2 and Huh-7 cells

The current investigation examined the impact of cisplatin, palbociclib, and their combined application on Huh-7 and HepG2 hepatocellular carcinoma cells. To quantify cell death, flow cytometry was employed. Huh-7 and HepG2 cells were initially cultured overnight in T-25 flasks. Subsequently, cells were treated for 48 h with cisplatin, palbociclib, a combination of both (C + P), or a control medium. Cells were detached using trypsin, washed with cold 1× phosphate-buffered saline (PBS), and centrifuged twice at 280×*g* for 5 min. The resulting cell pellets were then resuspended in ice-cold PBS and kept on ice until analysis. To distinguish between viable, early apoptotic, and late apoptotic/necrotic cells, a 100 µL aliquot of each cell suspension was stained with 1 µL propidium iodide (PI) solution (100 μg/mL) and 5 µL Annexin V-FITC, followed by a 15-min incubation in the dark at room temperature. After incubation, 400 µL of 1× Annexin binding buffer was added, and the samples were analyzed using a CytoFlex flow cytometer (Beckman Coulter, CA, USA) according to the manufacturer’s instructions. A minimum of 10^4^ events were recorded per sample, and data was processed using FlowJo software (Treestar Inc., San Carlos, CA, USA). This methodology allowed for a quantitative analysis of the effects of cisplatin, palbociclib, and their combination on the distribution of live, early apoptotic, and late apoptotic/necrotic cells within the Huh-7 and HepG2 cell populations.

### Cell cycle analysis

Flow cytometry was employed in this study to examine the impact of cisplatin, palbociclib, and their combined treatment (C + P) on cell cycle distribution. Four distinct treatment conditions, including cisplatin, palbociclib, combination therapy, and a control, were applied in 6-well plates. After a 48-h treatment, cells were collected, washed with ice-cold 1× PBS, and centrifuged twice at 280×*g* for 5 min. The resulting cell pellets were then resuspended in ice-cold PBS and kept at 4 °C until analysis. For flow cytometric analysis, cells were fixed in 2 mL of ice-cold 60% ethanol for 1 h. Following fixation, cells were thoroughly washed with PBS (pH 7.4) at least twice. The cell pellet was subsequently resuspended in a 1 mL solution of propidium iodide (PI, 10 μg/mL) and RNAase A (50 μg/mL) in PBS. This staining process, conducted at 37 °C for 20 min in the dark, allowed for the assessment of cellular DNA content. Subsequently, a CytoFlex flow cytometer (Beckman Coulter, CA, USA) was used to analyze the stained cells, adhering to the manufacturer’s protocol. A minimum of 104 events were acquired per sample. CytExpert software (Beckman Coulter, CA, USA) was then used to calculate the distribution of cells across the cell cycle phases. This entire procedure allowed for the quantification of the effects of cisplatin, palbociclib, and their combination on cell cycle progression within the treated cell populations.

### Data preprocessing

To retrieve both transcriptomic and clinical data for The Cancer Genome Atlas (TCGA) HCC samples, UCSC Xena platform was used to get TCGA DESeq2 normalized data (https://toil-xena-hub.s3.us-east-1.amazonaws.com/download/TCGA-GTEx-TARGET-gene-exp-counts.deseq2-normalized.log2.gz), as well as both TCGA clinical metadata (https://toil-xena-hub.s3.us-east-1.amazonaws.com/download/TcgaTargetGTEX_phenotype.txt.gz) and TCGA patients survival data (https://toil-xena-hub.s3.us-east-1.amazonaws.com/download/TCGA_survival_data). Next, data preprocessing steps were conducted to identify HCC sample IDs across TCGA gene expression counts following categorization based on HCC phenotype data. The resulting dataset included 424 TCGA HCC samples of both tumor (n = 365) and normal (n = 59) subtypes. Data curation and processing were performed using R programming language (v. 4.0). All pre-processed clinical data is provided in Supplementary Table S1.

### Survival analysis

To identify HCC patients’ survival in TCGA hepatocellular carcinoma dataset, samples were categorized into high and low levels of expression for PTEN, PI3K/AKT/mTOR pathway, β-catenin, BCL2, and HSP90 respectively. Then, using R packages “survival v.3.5” and “survminer v.0.4.9”, Kaplan–Meier (KM) plots were created using univariate cox regression to calculate the probability of survival for TCGA HCC patients based on TCGA HCC overall survival (OS) values, where high and low levels of studied genes and proteins is based on the median values of aforementioned HCC molecular markers.

### Statistical analysis

The study presented its findings primarily as average values with an indication of the inherent variability using standard error of the mean (SEM). Unless otherwise stated, one-way analysis of variance (ANOVA) was employed to assess statistical differences between multiple experimental groups. This was often followed by a post hoc Tukey–Kramer test to pinpoint specific group differences. A significance level of p less than or equal to 0.05 was considered statistically significant. GraphPad Prism (version 6.00, GraphPad Software, Inc., La Jolla, CA, United States) was used for all statistical analyses and data visualization. To ensure reproducibility, all experiments were repeated at least three times, each with three to six replicates per treatment condition. It is important to note that the presented data is representative of the overall findings.

## Results

### Cell viability (MTT) assay

**Fig. 1:**
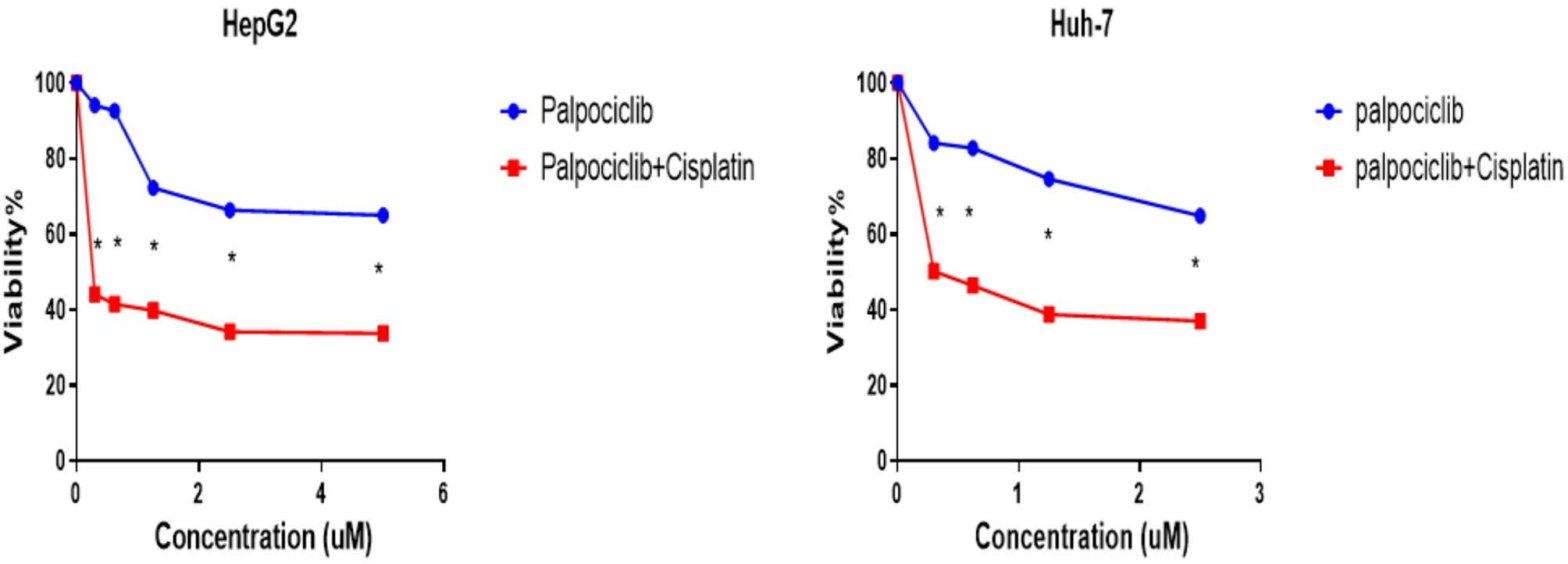
Cellular viability in HepG2 and Huh-7 liver cancer cells, after 48 h of treatment with palbociclib alone or in combination with cisplatin, was evaluated using an MTT assay. Statistical significance between treatments is indicated by: *p < 0.0001, determined via a Student’s Two-Tailed T-Test.

Initially, the individual efficacy of cisplatin and palbociclib against HepG2 and Huh-7 liver cancer cells was assessed. Cells were subjected to a range of drug concentrations over 48 h, and the IC50 values were determined for each drug alone. Subsequently, the combined effect of cisplatin and palbociclib was examined. HepG2 and Huh-7 cells were treated with a consistent cisplatin concentration (IC30) alongside varying palbociclib concentrations. This combined treatment significantly reduced the palbociclib concentration required for 50% growth inhibition (IC50) compared to palbociclib alone. Specifically, in HepG2 cells, the palbociclib IC50 decreased from 1.016 to 0.293 µM, and in Huh-7 cells, from 1.33 to 0.46 µM. To quantify the drug interaction, a combination index was calculated. A combination index below 1 indicates synergism, where the combined effect surpasses the sum of individual drug effects. For HepG2 cells, the combination index was 0.617, confirming a synergistic interaction. However, in Huh-7 cells, the combination index was 0.8, suggesting an additive effect, where the combined treatment effect was comparable to the sum of individual drug effects (as illustrated in Fig. 1 and Table 2).

**Table 2:** The results are presented as calculated IC50 values (concentration inhibiting 50% of cell growth) for both Palbociclib alone and Palbociclib combined with cisplatin. Additionally, combination indices are included to reveal the interaction between the drugs in HepG2 and Huh-7 cells. These indices likely indicate a synergistic effect, meaning the combined treatment is more effective than the sum of the individual drugs, in HepG2 cells. On the other hand, the data suggests an additive effect in Huh-7 cells, where the combination acts like the effects of each drug added together.

Drug	IC50 uM	Combination index
HepG2 cell line		
Palbociclib	1.016	0.617 (synergistic)
Palbociclib + cisplatin	0.2938	
Huh-7 cell line		
Palbociclib	1.336	0.8 (additive)
Palbociclib + cisplatin	0.463	

### Scratch wound assay

**Fig. 2:**
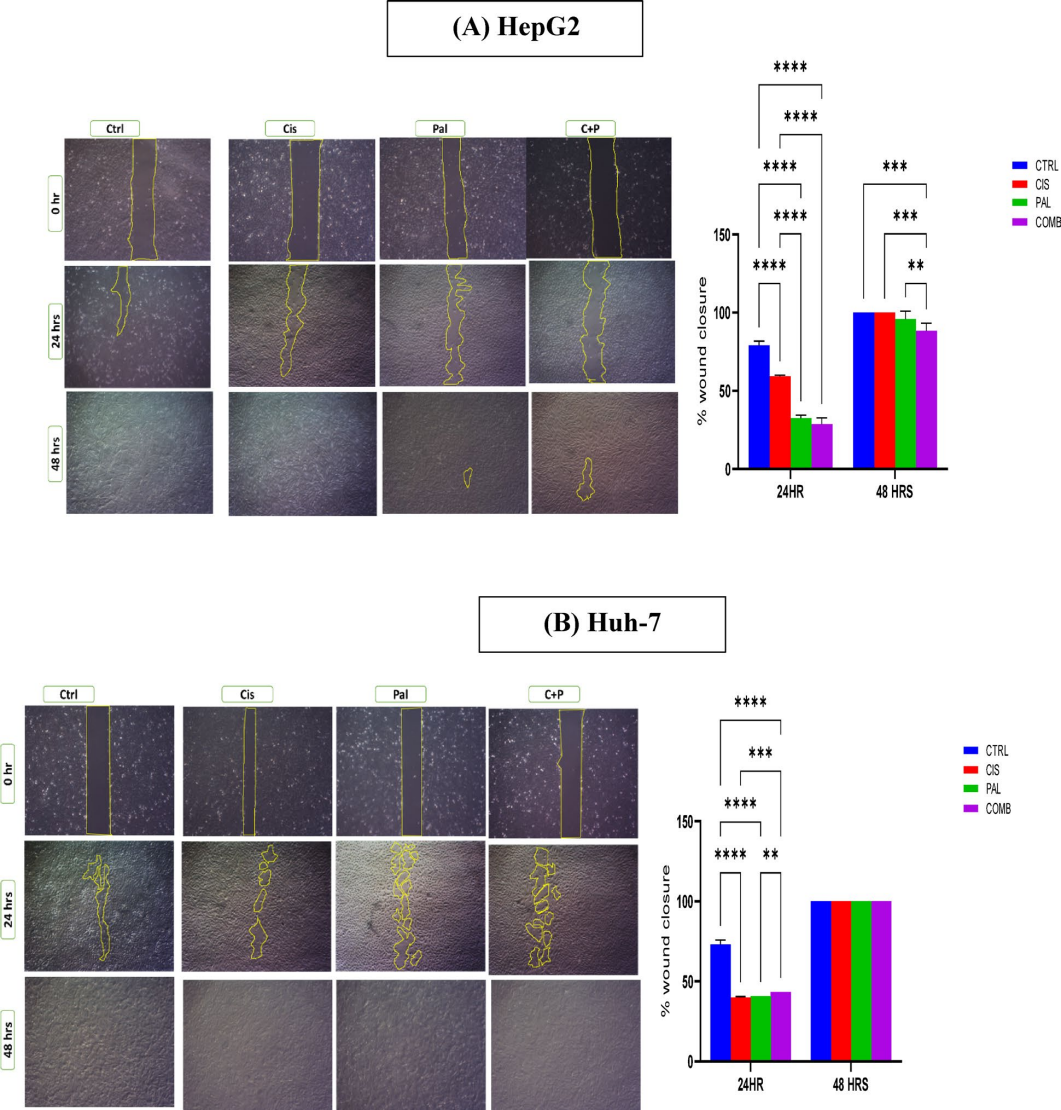
The impact of drug treatments on HepG2 and Huh-7 cell migration was evaluated using a scratch wound assay. Photomicrographs, capturing the wound closure at 0-, 24-, and 48-h post-treatment, are presented for both HepG2 (**A**) and Huh-7 (**B**) cells. ImageJ software was employed to quantify wound closure by analyzing the photomicrographs and calculating the percentage of closure. Statistical analysis, using a two-way ANOVA with Tukey’s post hoc test, was conducted to compare wound closure between treated and untreated control groups at each time point. A minimum of three wells per treatment (n = 3 wells/treatment) were analyzed. Statistical significance, when compared to the untreated control at corresponding time points, is indicated as: +; ***p = 0.0001; and **p < 0.001.

Epithelial to mesenchymal transition (EMT) represents cell migration and metastasis through several steps as loss of cell adhesion molecules and cellular polarity^29^ Hence, we investigated HCC cells undergoing EMT using scratch wound assay. The data obtained represented the ability of cisplatin, and palbociclib as single treatments or combined to alter HepG2 and Huh-7 cell metastasis. At the 24-h mark, the data revealed a significant reduction in wound closure, indicative of decreased metastasis, in HepG2 cells treated with palbociclib alone (32.49%) compared to untreated controls (79.07%). Combining palbociclib with cisplatin further enhanced this reduction, with a wound closure percentage of (28.74%) compared to cisplatin alone (59.34%). At the 48-h mark, both cisplatin and control wells exhibited complete wound closure, while palbociclib and C + P treated wells displayed a decreased in wound closure compared to untreated control and cisplatin wells (95.92% and 88.27%, respectively) (as illustrated in Fig. 2A).

Regarding Huh-7 cell line, cells treated with cisplatin, palbociclib, and the combination all showed significant reductions in wound closure percentages (39.91%, 40.83%, and 43.3%, respectively) at 24 h when compared to untreated control (73.07%). However, all groups displayed unexpected complete wound closure within 48 h (as illustrated in Fig. 2B). These findings suggest that palbociclib, either alone or combined with cisplatin, may partially delay the metastasis and migration of HCC-derived cells, although the observed later increase in wound closure warrants further investigation.

### Using annexin V/propidium iodide staining to detect apoptosis in HepG2 and Huh-7 cells

Flow cytometry and Annexin V/PI staining were utilized to investigate the contribution of apoptosis to the cytotoxic effects of the administered drugs. The analysis focused on the percentage of apoptotic cells within the HepG2 and Huh-7 cell lines following treatment with cisplatin, palbociclib (as single agents and combined), and an untreated control.

The results of HepG2 regarding the alive cell population revealed a significant difference (*p* > 0.0001) between all groups. The untreated control exhibited the highest proportion of viable cells (67.7%). Conversely, all drug treatments caused a significant decrease in the live cell population compared to the control. Interestingly, cisplatin treatment induced the most prominent increase in early apoptosis (1.17%) compared to control (*p* = 0.007). However, it also induced an increase in both late apoptosis (6.52%) and necrosis (43.0%) compared to control (*p* < 0.0001). This observation suggests a potential distinction in the cell death pathway triggered by cisplatin compared to the other treatments.

**Fig. 3:**
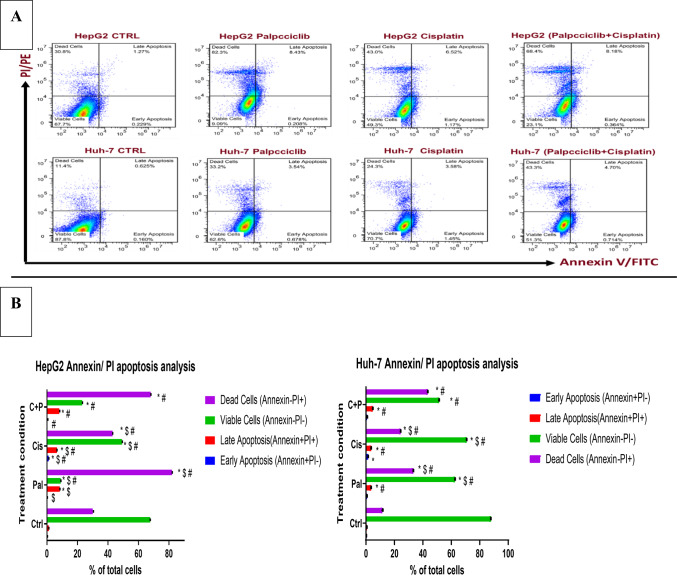
The effect of cisplatin, palbociclib (alone and in combination), on the proportion of apoptotic cells in HepG2 and Huh-7 liver cancer cell lines was evaluated using an Annexin V/PI apoptosis assay. The data is presented in two formats: (**A**) Dot Plots: Representative dot plots illustrate the distribution of cells based on Annexin V-FITC and PI signals, which categorize cells into apoptotic stages. (**B**) Bar Chart: This chart displays the cell population distribution across different apoptosis stages: viable, early apoptotic, and late apoptotic/necrotic. Statistical analysis, using one-way ANOVA with Tukey’s post hoc test, showed a significant difference (*p* < 0.0001) in the percentage of viable cells across all treatments for both HepG2 and Huh-7 cell lines. Statistical significance is denoted as follows: “*p < 0.05” signifies a difference relative to the untreated control, “^$^*p* < 0.05” indicates a difference compared to single-agent treatment, and “^#^*p* < 0.05” represents a difference compared to combination therapy. All comparisons were conducted using one-way ANOVA with Tukey’s post hoc test, with a minimum of three wells per treatment (n = 3 wells/treatment).

Palbociclib, on the other hand, displayed the most potent cell death effect. It yielded the lowest percentage of live cells (9.09%) and the highest levels of both late apoptosis (8.43%) and necrosis (82.3%) amongst all groups (*p* < 0.0001) followed by the combination treatment (C + P) that also significantly reduced the live cell population (23.1%) and caused a substantial increase in late apoptosis (8.18%) and necrosis (68.4%) when compared to cisplatin monotherapy and control (as illustrated in Fig. 3).

The results for Huh-7 cells were similar to those seen in HepG2 cells. In the untreated control group, a high percentage of cells (87.8%) remained viable. All drug treatments significantly reduced the viability compared to control (*p* > 0.0001). Cisplatin alone caused a moderate decrease in living cells (70.7%), while palbociclib alone and the combination (C + P) caused a more substantial decrease (62.6% and 51.3% alive, respectively). Interestingly, cisplatin monotherapy induced a significant increase in early apoptosis (1.45%) compared to control (*p* = 0.0031), suggesting it triggers programmed cell death to a certain extent. However, both Palbociclib alone and the C + P combination had a more pronounced effect on late apoptosis (3.58% and 4.7%, respectively) and necrosis (24.3% and 43.3%, respectively) compared to control (*p* < 0.0001). This indicates these treatments cause a greater shift towards late-stage cell death and necrosis compared to cisplatin alone. Overall, the combination therapy (C + P) resulted in the highest percentage of cells undergoing late apoptosis and necrosis, followed by palbociclib alone and then cisplatin alone. Conversely, cisplatin monotherapy led to the highest percentage of cells in early apoptosis, while the combination treatment had the lowest proportion of viable cells. Hence, these findings demonstrated that both cisplatin and palbociclib treatments induce apoptosis and necrosis in Huh-7 liver cancer cells, with the combination treatment potentially exhibiting the strongest cell death effect. Notably, cisplatin consistently induced a slight increase in early apoptosis in both cell lines, possibly indicating a distinct cell death pathway compared to palbociclib and the combination therapy (as illustrated in Fig. 3).

### Cell cycle analysis

Flow cytometry analysis of HepG2 cells revealed distinct cell cycle effects following treatment with cisplatin, palbociclib alone and combined, compared to the untreated control (73.41% G0/G1, 23.6% S, 2.9% G2/M). Palbociclib significantly increased the G0/G1 population (89.8%, *p* < 0.0001) while significantly decreasing both S (9.3%, *p* < 0.0001) and G2/M (0.95%, *p* < 0.0001) phases. Conversely, cisplatin treatment caused a significant decrease in G0/G1 (21.11%, *p* < 0.0001) with a significant increase in S phase (76.02%, *p* < 0.0001), suggesting S-phase arrest. No significant change was observed in the G2/M phase (2.85%) compared to control. The combination treatment (C + P) displayed a decrease in both G0/G1 (63.8%, *p* < 0.0001) and G2/M (1.57%, *p* < 0.0001) phases, along with a significant increase in S phase (34.20%, *p* < 0.0001), indicating potential targeting of multiple cell cycle checkpoints.

Analysis of Huh-7 cells using flow cytometry yielded similar cell cycle distribution patterns to the HepG2 cells. The untreated control group displayed a distribution of 87.1% in G0/G1, 10.9% in S phase (DNA synthesis), and 1.8% in G2/M (mitosis) phases. Palbociclib treatment did not cause significant changes in either the G0/G1 (87.8%) or S phase (10.86%) compared to control. However, it did induce a significant decrease in the G2/M phase (1.26%, *p* < 0.01), suggesting a similar G0/G1 arrest effect observed in HepG2 cells. In contrast, cisplatin treatment resulted in a significant decrease in the G0/G1 phase (16.71%, *p* < 0.0001) and a substantial increase in both the S phase (79.91%, *p* < 0.0001) and G2/M phase (3.36%, *p* < 0.0001) compared to control. This pattern points towards a potential S-phase arrest induced by cisplatin in Huh-7 cells. The combination treatment (C + P) mirrored the effects seen in HepG2 cells. It caused a significant decrease in both the G0/G1 phase (56.6%, *p* < 0.0001) and the G2/M phase (1.57%, *p* < 0.01), accompanied by a significant rise in the S phase (42.01%, *p* < 0.0001). This suggests that the combination therapy might target multiple cell cycle checkpoints in Huh-7 cells as well.

**Fig. 4:**
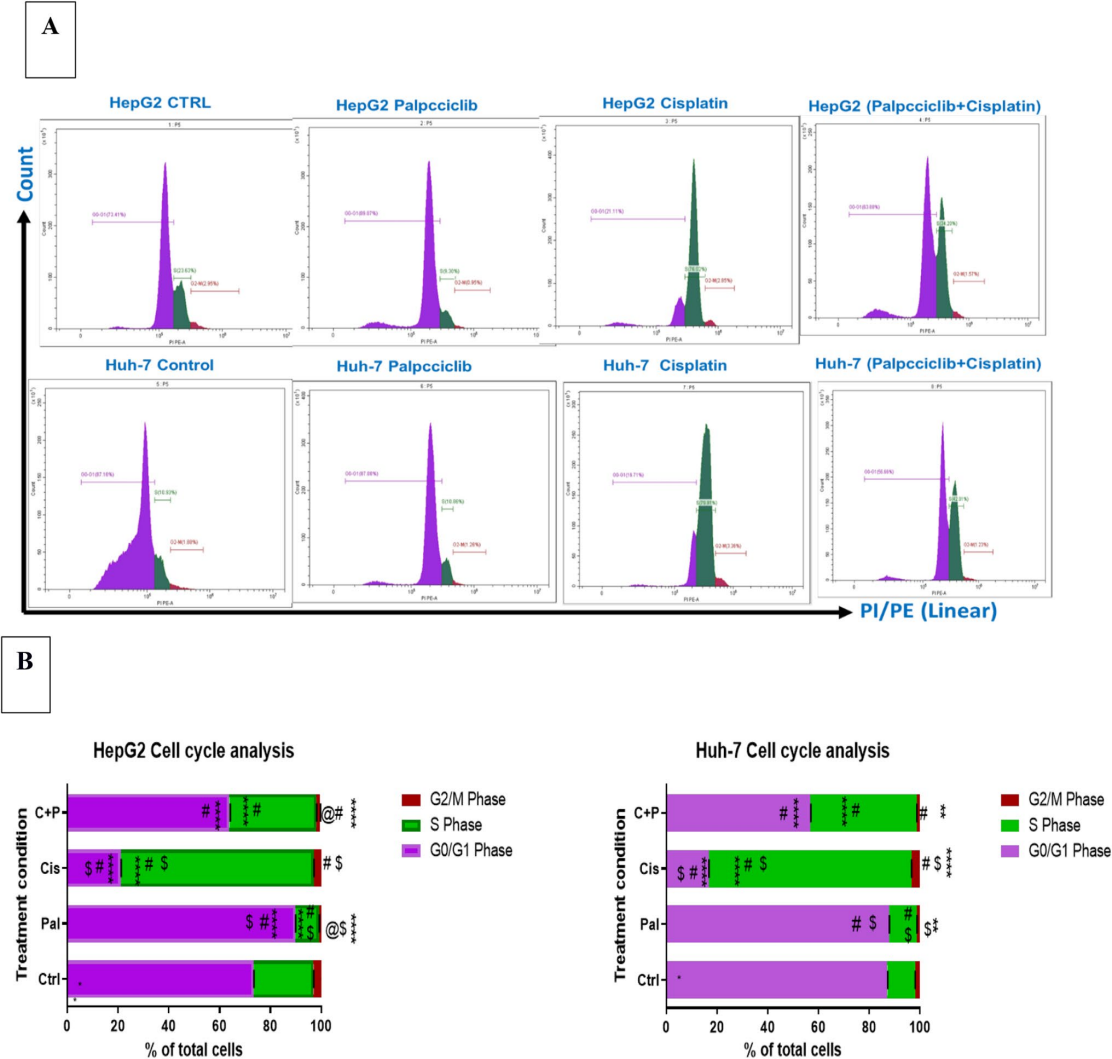
The influence of cisplatin, palbociclib (administered alone or in combination), on cell cycle progression in HepG2 and Huh-7 liver cancer cells was assessed through flow cytometric analysis. The data is presented in two formats: (**A**) Histograms: Representative histograms illustrate the cell distribution across different cell cycle phases (G0/G1, S-phase, G2/M) for each treatment condition. (**B**) Stacked Bar Chart: This chart quantifies the percentage of cells in each cell cycle phase (G0/G1, S-phase, G2/M) for each treatment group. Statistical analysis, using one-way ANOVA with Tukey’s post hoc test (n = 3 wells/treatment), was performed. The following significance levels were observed: ****p < 0.0001: Highly significant difference compared to the untreated control. ^$^*p* < 0.0001: Extremely significant difference between palbociclib and cisplatin monotherapy groups. ^#^*p* < 0.0001: Very significant difference between monotherapy groups and the combination therapy (C + P). ^@^*p* = 0.0091: Significant difference between monotherapy groups and C + P, though less pronounced than #. These results indicate that the treatments impact cell cycle progression in both HepG2 and Huh-7 cells.

The study revealed distinct cell cycle arrest patterns in both HepG2 and Huh-7 cell lines following treatment with cisplatin and the combination therapy (C + P). These treatments resulted in a higher number of cells arrested in the S-phase compared to the untreated control group. This effect appears to be primarily driven by cisplatin, as both cisplatin alone and C + P displayed a significantly increased percentage of cells in the S-phase compared to palbociclib monotherapy (*p* < 0.0001). In HepG2 cells, the percentage of S-phase cells reached 76.02% for cisplatin and 34.20% for C + P, while in Huh-7 cells, it was 79.91% for cisplatin and 42.01% for C + P. These findings are visually represented in Fig. 4.

### RT-qPCR results of PI3K/AKT/mTOR pathways, B-catenine, BCL2 and Hsp90

**Fig. 5:**
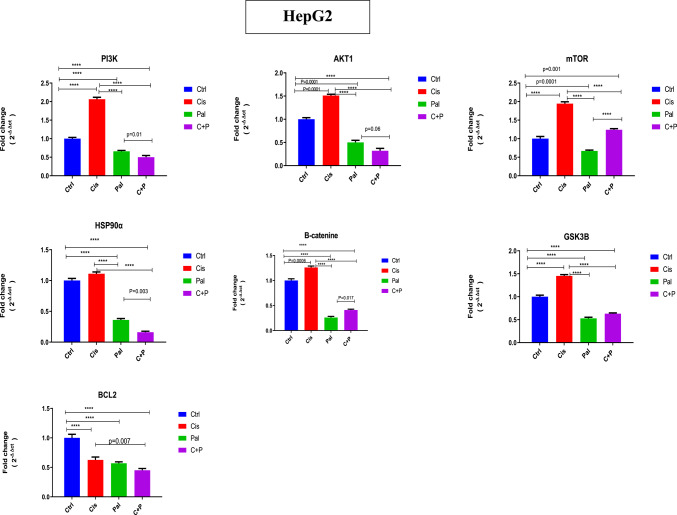
The impact of drug combinations on the PI3K/AKT/mTOR pathway, B-catenin, BCL2, and Hsp90 in HepG2 liver cancer cells was evaluated. RT-qPCR was used to quantify the fold change in mRNA levels of relevant genes, with calculations performed using the 2^−ΔΔCT^ method. Palbociclib and the C + P combination significantly downregulated Hsp90, Gsk3B, B-catenin, and PI3K/AKT/mTOR pathway-related genes compared to cisplatin alone and the untreated control. Conversely, cisplatin alone significantly upregulated B-catenin, Gsk-3B, and PI3K/AKT/mTOR pathway genes relative to palbociclib, the C + P combination, and the control. For Hsp90, PI3K, and AKT, the C + P combination exhibited the greatest reduction, followed by palbociclib. Palbociclib alone showed the most pronounced reduction in Gsk3B, B-catenin, and mTOR, followed by the C + P combination. All three treatments (palbociclib, cisplatin, and C + P) significantly reduced oncogenic BCL2 expression compared to the untreated control. ****p < 0.0001, and other significant *p* values are specified in the figure, as determined by one-way ANOVA with Tukey’s post hoc test.

Analysis of mRNA expression via RT-qPCR demonstrated that palbociclib and the combined treatment (C + P) significantly reduced the expression of key genes involved in several hepatocellular carcinoma (HCC) pathways in HepG2 cells, relative to cisplatin monotherapy and the untreated control. These pathways included heat shock protein 90 (HSP90), glycogen synthase kinase 3 beta (GSK3B), and the PI3K/AKT/mTOR pathway, which is implicated in cell growth and survival. Conversely, cisplatin treatment significantly elevated gene expression within these same pathways (Hsp90, B-catenin, Gsk-3B, and PI3K/AKT/mTOR) when compared to palbociclib monotherapy, the C + P combination, and the untreated control. For Hsp90, PI3K, and AKT, the C + P combination exhibited the most pronounced reduction, followed by palbociclib monotherapy. Regarding Gsk3B, B-catenin, and mTOR, palbociclib monotherapy showed the greatest reduction, followed by the C + P combination. Palbociclib, cisplatin monotherapy, and the C + P combination all significantly decreased the expression of the oncogenic BCL2 compared to the untreated control (Fig. 5).

**Fig. 6:**
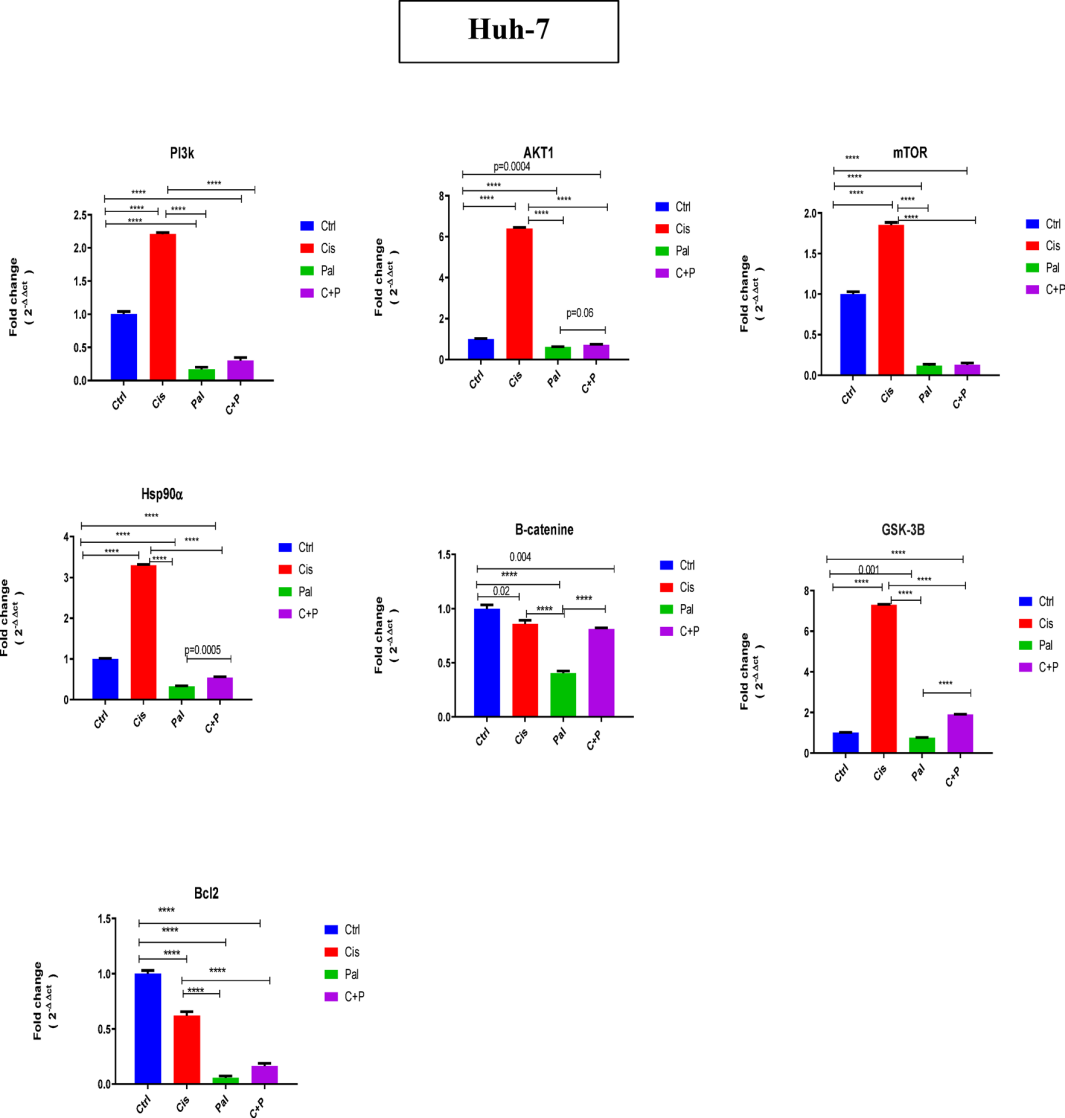
The influence of drug combinations on the PI3K/AKT/mTOR pathway, B-catenin, GSK3B, BCL2, and Hsp90 in Huh-7 liver cancer cells was examined. RT-qPCR was employed to quantify mRNA fold changes for genes within these pathways, specifically PI3K, AKT, mTOR, B-catenin, GSK3B, BCL2, and Hsp90. In Huh-7 cells, both palbociclib and the C + P combination significantly decreased the expression of Hsp90, GSK3B, and PI3K/AKT/mTOR pathway genes, relative to cisplatin monotherapy and the untreated control. Conversely, cisplatin monotherapy significantly increased the expression of these genes compared to palbociclib, the C + P combination, and the control. All three treatments (palbociclib, cisplatin, and C + P) significantly reduced the expression of oncogenic BCL2 and B-catenin relative to the control. ****p < 0.0001, with additional significant *p* values detailed in the figure, as determined by one-way ANOVA with Tukey’s post hoc test.

In Huh-7 cells, palbociclib and the C + P combination significantly decreased the expression of Hsp90, Gsk3B, and PI3K/AKT/mTOR pathway genes, compared to cisplatin monotherapy and the untreated control. Conversely, cisplatin monotherapy significantly increased the expression of these same genes when compared to palbociclib, the C + P combination, and the untreated control. Palbociclib, cisplatin, and the C + P combination all significantly reduced the expression of the oncogenic BCL2 and B-catenin relative to the untreated control. Palbociclib monotherapy exhibited the most substantial reduction, followed by the C + P combination, and then cisplatin monotherapy (Fig. 6).

### Western blot results regarding PTEN and PAKT protein expression

**Fig. 7:**
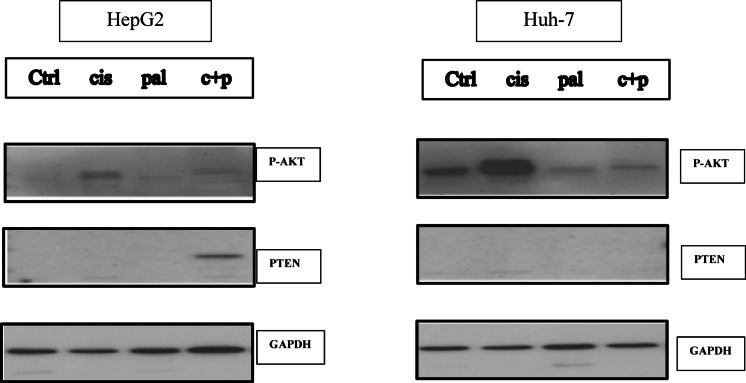
Protein expression of PTEN and P-AKT was evaluated using Western blot analysis, with GAPDH serving as a normalization control. The combined treatment of palbociclib and cisplatin resulted in a significant enhancement of cisplatin’s antitumor effects, as evidenced by a marked reduction in phosphorylated Akt (p-Akt) levels in both HepG2 and Huh-7 cells. Interestingly, PTEN protein expression was increased only in HepG2 cells following combination therapy, indicating a possible cell line-dependent mechanism that may contribute to the observed antitumor effects.

Analysis of phosphorylated Akt (p-Akt) levels, an indicator of Akt activation, revealed distinct patterns across treatment groups. While cisplatin treatment significantly increased p-Akt levels in both HepG2 and Huh-7 cells, consistent with enhanced Akt signaling, both palbociclib and the combination treatment resulted in a significant downregulation of p-Akt levels compared to the cisplatin-treated groups in both cell lines. In contrast, in HepG2 cells, palbociclib and the combination treatment did not significantly alter p-Akt levels compared to the control group. GAPDH, utilized as an internal control for normalization, exhibited consistent expression across all groups, validating its suitability for data normalization. Notably, PTEN protein expression was exclusively detected in the HepG2 cells treated with the combination therapy, suggesting a potential cell line-specific upregulation of PTEN expression upon combined treatment (see Fig. 7, Supplementary Fig. 1).

### Survival analysis shows PTEN, PI3K/AKT/mTOR pathway, β-catenin/BCL2 and β-catenin/HSP90 as prognostic factors for survival of TCGA HCC patients

**Fig. 8:**
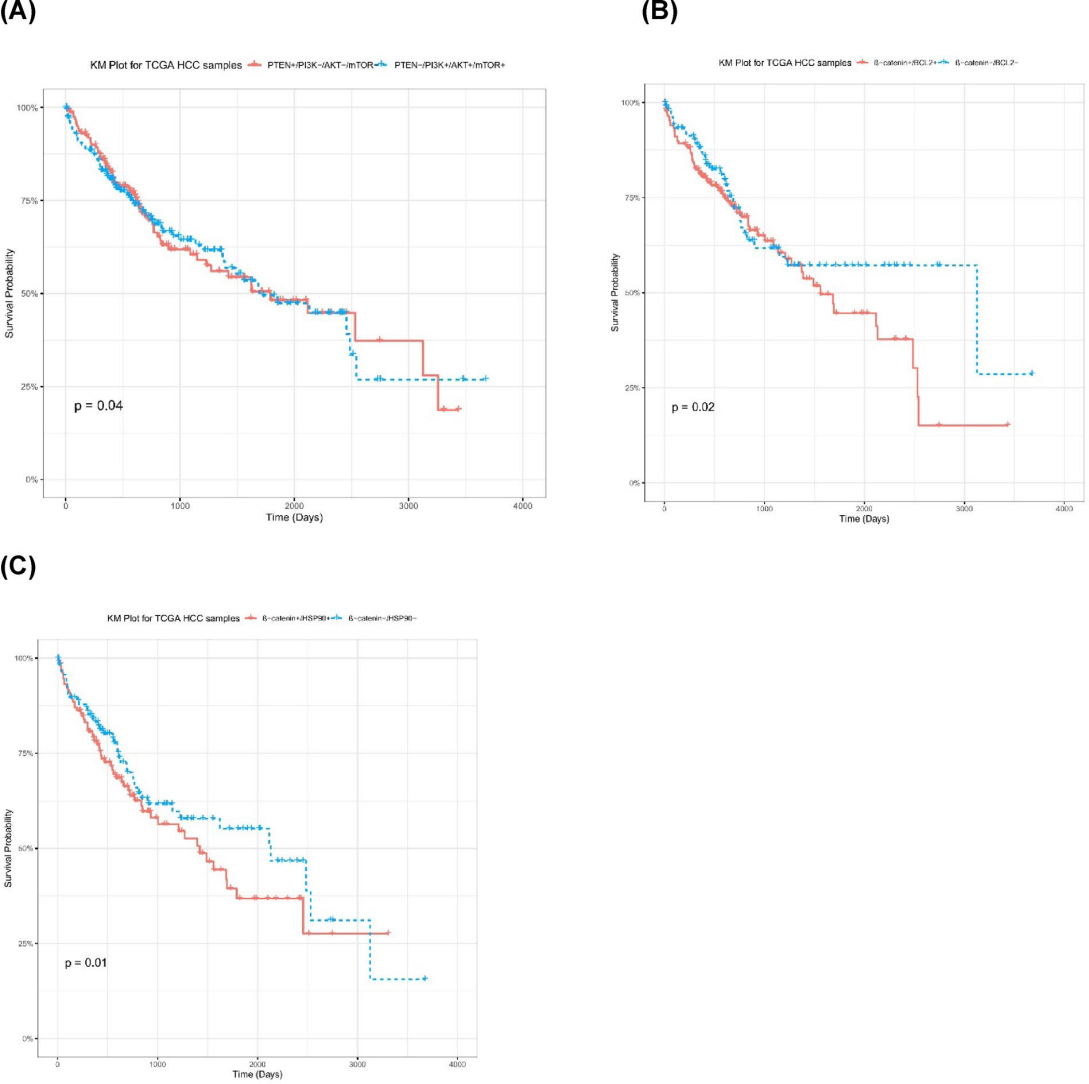
Survival analysis for high/low molecular markers in TCGA HCC samples. (**A**) KM plot for TCGA HCC samples (PTEN+/PI3K−/AKT−/mTOR− vs. PTEN−/PI3K+/AKT+/mTOR+). (**B**) KM Plot for TCGA HCC samples (β-catenin+/BCL2+ vs. β-catenin−/BCL2−). (**C**) KM plot for TCGA HCC samples (β-catenin+/HSP90+ vs. β-catenin−/HSP90−).

HCC data was assessed using survival analysis to measure PTEN, PI3K/AKT/mTOR pathway, β-catenin/BCL2, and β-catenin/HSP90 effect on prognosis of hepatocellular carcinoma patients using Kaplan–Meier (KM) plots. All HCC samples were classified based on median expression for studied markers. Analysis showed that TCGA HCC samples with high PTEN levels and low PI3K/AKT/mTOR pathway levels (PTEN+/PI3K−/AKT−/mTOR−) had better survival probability compared to samples with low PTEN and high PI3K/AKT/mTOR pathway levels (PTEN−/PI3K+/AKT+/mTOR+) (*p* = 0.04) (Fig. 8A). This provides evidence that PTEN can be used as modulator for PI3K/AKT/mTOR pathway and leads to enhanced patients’ survival. Also, Kaplan–Meier plot for samples with both low β-catenin and BCL2 levels (β-catenin−/BCL2−) showed significantly better survival probability compared to samples with high levels of both markers (β-catenin+/BCL2+) (*p* = 0.02) (Fig. 8B). Moreover, HCC samples with low levels of β-catenin and HSP90 (β-catenin−/HSP90−) showed consistently higher probability for survival of HCC patients compared to samples with high levels of both β-catenin and HSP90 (β-catenin+/HSP90+) (*p* = 0.01) (Fig. 8C). This proves that β-catenin is a key modulator of HCC survival following interaction with both BCL2 and HSP90. Kaplan–Meier curves for TCGA HCC samples with (β-catenin+/BCL2−) levels compared to (β-catenin−/BCL2+), as well as samples with (β-catenin+/HSP90−) levels compared to (β-catenin−/HSP90+) are provided in Supplementary Fig. 2.

## Discussion

Cancer combination therapy seeks to achieve two primary goals: amplifying and extending the therapeutic benefits of single-drug treatments while simultaneously managing side effects and delaying the emergence of resistance. This approach has become increasingly feasible due to the development of novel drugs with unique mechanisms of action. These drugs often target specific molecules within cancer cells and have distinct side effect profiles compared to traditional chemotherapeutic agents. Combining these novel drugs with established therapies, even those like cisplatin that are considered “classical” chemotherapeutic drugs, represents a significant advancement in cancer treatment.

Despite their efficacy, cisplatin and its class Platinum-based drugs represent a widely used and effective cancer treatment due to their broad efficacy across diverse malignancies^30^. However, this therapeutic class is unfortunately limited by a dose-limiting toxicity profile encompassing both general cytotoxicity and platinum-specific organ toxicities, particularly affecting the kidneys and nervous system^31^.

Cisplatin remains a fundamental chemotherapeutic drug, often integrated into combination therapies to maximize clinical effectiveness. Nevertheless, there’s a persistent demand to optimize these combinations for better patient results. Preclinical findings indicate potential synergistic effects between cisplatin and CDK4/6 inhibitors in several cancers, such as esophageal squamous cell carcinoma, ovarian cancer, bladder cancer, and breast cancer. However, the combined efficacy of cisplatin and palbociclib, a selective CDK4/6 inhibitor, has not been thoroughly investigated in hepatocellular carcinoma (HCC). This lack of comprehensive study points to a significant knowledge gap and emphasizes the importance of focused research into this potential treatment approach for HCC^32–34^.

The current study examined the combined effects of cisplatin and palbociclib on HepG2 and Huh-7 human HCC cells in vitro. MTT assays indicated a synergistic response in HepG2 cells and an additive response in Huh-7 cells. This observation is consistent with Rossini et al.^35^, who documented predominantly additive or synergistic interactions between palbociclib and cisplatin in human testicular germ cell tumor cell lines.

Furthermore, our findings on the synergistic interaction between cisplatin and palbociclib are further supported by a study by Xiang et al.^36^, Which explored the synergistic effects of cisplatin and palbociclib in treating triple-negative breast cancer (TNBC) using a novel nanoscale drug delivery system.

In the scratch assay, the initial results were promising, showing a significant reduction in wound closure compared to untreated controls after 24 h. However, this effect diminished over time, with most wells exhibiting near-complete wound closure by the second day. Only the combination treatment in HepG2 cells showed persistent inhibition (61% wound closure).

It was proposed that the palbociclib metabolism by the liver, primarily via CYP3A4 (supported by Shah et al.^37^), was responsible for the reduced efficacy. This is further supported by Choi et al.^38^, who reported higher CYP3A4 expression in Huh7 cells compared to HepG2. Besides, Qin et al.^39^ showed Palbociclib’s potential in reducing metastasis through the c-Jun/COX signaling pathway. Supporting this study, other research has demonstrated palbociclib’s ability to inhibit epithelial-to-mesenchymal transition (EMT) in cancer^40,41^. Additionally, other studies highlight the potential of palbociclib, alone or in combination, for its antimetastatic properties based on reduced wound healing and Wnt/β-catenin gene expression which was linked to metastasis as supported by Gupta et al.^42,43^.

Contrary to the MTT assay results, the Annexin V/PI apoptosis assay revealed different trends. In HepG2 cells, palbociclib alone induced the highest overall percentage of apoptotic cells (both early and late), and the lowest percentage of viable cells, followed by the C + P combination and then cisplatin alone. In Huh-7 cells, the C + P combination showed the highest percentage of apoptotic cells, followed by cisplatin alone and then palbociclib alone.

The observed increase in apoptosis aligns with findings reported by Brown et al.^30^, who demonstrated that the combination of palbociclib and cisplatin significantly enhanced apoptosis in the MDA-MB-231 triple-negative breast cancer cell line compared to cisplatin alone.

Flow cytometric analysis of the cell cycle in HepG2 and Huh-7 cell lines showed differential responses to cisplatin, palbociclib alone, and the combined treatment (C + P). Both cisplatin and the combination therapy, relative to the control and palbociclib alone, resulted in a significantly increased proportion of cells arrested in the S-phase, with cisplatin demonstrating the predominant effect. Furthermore, cisplatin and the combination led to a higher percentage of cells in the G2/M phase compared to palbociclib alone. Conversely, palbociclib alone and the C + P combination induced an elevation in the G0–G1 phase population compared to cisplatin alone. Notably, palbociclib alone exhibited a greater percentage of cells in G0–G1 than the control.

The combined treatment with both drugs resulted in a significant arrest of cells in two key cell cycle phases: S-phase and G0/G1. This suggests that both drugs likely play a role in the overall cell death effect (cytotoxic effect) observed when they are used together. The cells were able to bypass the initial G1 arrest caused by palbociclib were then subsequently blocked by Cisplatin during the S-phase. This two-pronged attack on the cell cycle progression might contribute to the additive or synergistic effect seen in our experiments.

The observed cell cycle alterations align with Huang et al.^44^, who reported an increase in the G0–G1 phase population and a decrease in G2 and S phase cells following palbociclib treatment in a breast cancer cell line. Additionally, Wang et al.^45^ documented a distinct S-phase cell cycle arrest induced by cisplatin in HepG2 cells. However, other studies have shown that cisplatin and the C + P combination induce cell cycle arrest at the G2/M phase^46,47^. The observed discrepancies in cell cycle arrest patterns between experiments could be attributed to several factors. Firstly, inherent differences between the employed cell lines (HepG2 and Huh-7) might influence their response to treatment. Secondly, the dosages of cisplatin used and the duration of exposure could significantly impact the cell cycle arrest profile. Cisplatin primarily might induce S-phase arrest during the early stages of treatment when cells experience stress due to DNA damage. This initial S-phase arrest could represent a direct cellular response to the DNA replication disruption caused by cisplatin. However, to fully understand the dynamics of cell cycle changes following cisplatin treatment, a more comprehensive investigation is necessary. This investigation should explore the effects of various factors, including cell line type, drug dosage, and exposure time, on the cell cycle profile^45^.

To investigate potential mechanisms underlying the observed synergy between the investigated drugs, we focused on key HCC-associated signaling pathways known to be dysregulated in hepatocellular carcinoma. These pathways, PI3K/AKT/mTOR and Wnt/β-catenin, are reportedly upregulated by Hsp90 and downregulated by PTEN^48,49^, further highlighting their potential involvement in HCC development. Moreover, aberrant activation of the PI3K/AKT/mTOR pathway has been linked to cisplatin resistance. By targeting this pathway with both palbociclib and cisplatin, the combination therapy might overcome this resistance and enhance overall tumor suppression. We hypothesized that the combined effect on the PI3K/AKT/mTOR pathway could be synergistic, leading to a more potent inhibition of cell proliferation and survival signals, ultimately resulting in superior tumor control. Therefore, we employed RT-qPCR and western blot analysis to evaluate mRNA and protein expression levels respectively and assess the effects of the drug combination on these critical pathways in Huh-7 and HepG2 cells.

Our findings demonstrate that the combination of palbociclib and cisplatin effectively inhibited Akt signaling in both HepG2 and Huh-7 cells, as evidenced by the significant downregulation of phosphorylated Akt (p-Akt) levels compared to cisplatin treatment alone. This observation aligns with the known anti-proliferative effects of CDK4/6 inhibitors and supports their potential therapeutic utility in cancers with aberrant Akt signaling. Furthermore, the observed downregulation of key genes involved in cell survival and proliferation, including Hsp90, Gsk3β, B-catenin, and components of the PI3K/Akt/mTOR pathway, provides strong evidence for the anti-proliferative and pro-apoptotic effects of these treatments. Notably, the combination therapy exhibited the most pronounced effects in HepG2 cells, evidenced by the strongest reduction in Hsp90, PI3K, and Akt expression levels. This coincided with a unique observation: PTEN protein expression was exclusively detected in HepG2 cells treated with the combination therapy, suggesting a potential cell line-specific mechanism contributing to the observed antitumor effects.

Palbociclib exerts anti-metastatic effects beyond cell cycle arrest. Qin et al.^39^ demonstrated that palbociclib disrupts the c-Jun/COX-2 signaling pathway, leading to downregulation of vimentin and Snail, key regulators of epithelial-to-mesenchymal transition (EMT). This inhibition of EMT suppresses cancer cell motility and invasion. Furthermore, palbociclib suppresses COX-2 expression and PGE2 production, hindering c-Jun-mediated COX-2 activation. Notably, Horpratraporn et al.^50^ observed that palbociclib suppresses Akt and Snail, further supporting its antimetastatic potential.

Importantly, COX-2 is known to activate the PI3K/Akt pathway, a critical regulator of cell survival and a known contributor to cisplatin resistance in various cancers^51,52^. By inhibiting COX-2, palbociclib may indirectly suppress the PI3K/Akt pathway, potentially sensitizing cancer cells to cisplatin. This suggests that combining palbociclib with cisplatin may enhance therapeutic efficacy by targeting multiple pathways involved in tumor growth and survival.

**Fig. 9:**
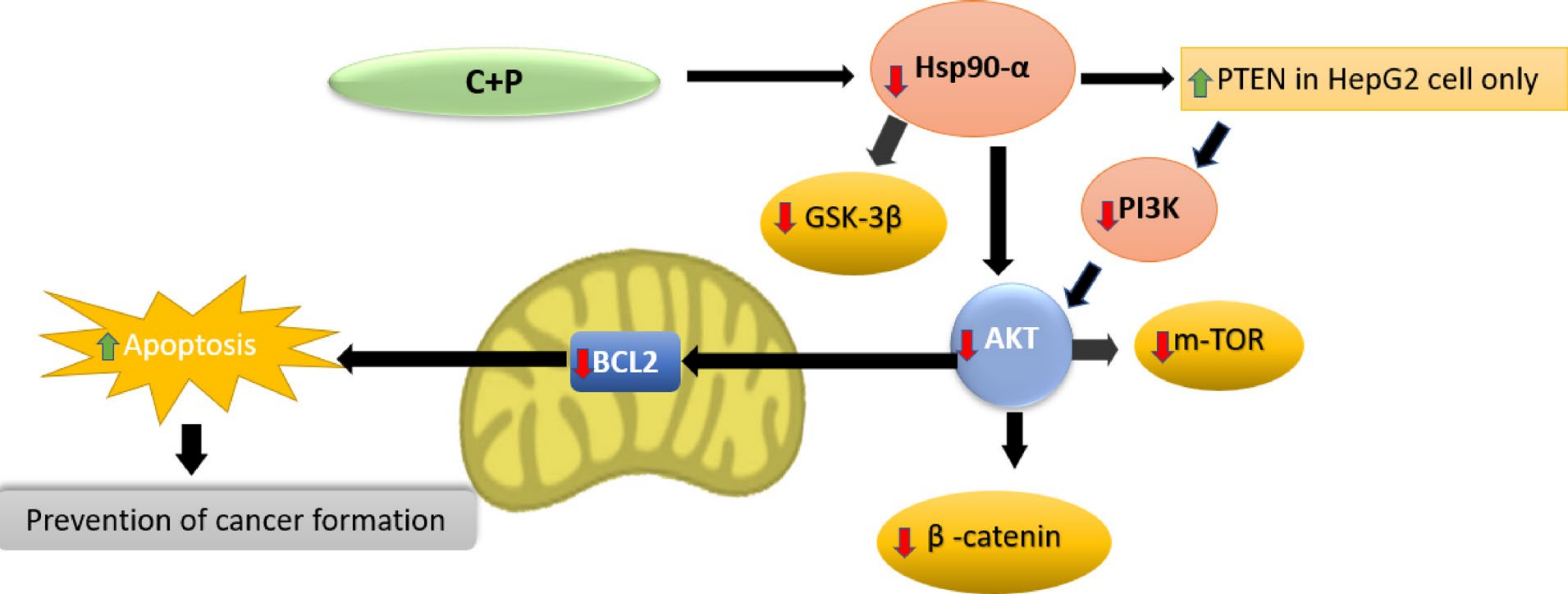
Proposed molecular mechanisms of palbociclib and cisplatin combination therapy in HepG2 and Huh-7 cell lines. This diagram illustrates the proposed molecular mechanisms underlying the antitumor effects of the palbociclib and cisplatin combination therapy in HepG2 and Huh-7 cells. The combination therapy exerts its effects through a multifaceted approach.

In addition to the direct effects of the drugs, the altered expression of the studied target genes may also be influenced by their interconnected regulatory networks (Fig. 9). For instance, recent research has demonstrated that Akt-mediated phosphorylation of β-catenin enhances its transcriptional activity and promotes tumor cell invasion^53^, highlighting the significance of Akt-dependent regulation of β-catenin in tumor progression. Furthermore, studies have shown that Akt upregulates Bcl-2, an anti-apoptotic protein that is often overexpressed in cisplatin-resistant cancers^54^. This observation supports the notion that targeting Akt signaling pathways, as achieved by the combination therapy, may overcome cisplatin resistance.

Mechanistically, the combination therapy may exert its strongest impact in HepG2 cells by synergistically inhibiting Hsp90, a molecular chaperone crucial for stabilizing several oncogenic proteins, including those involved in the PI3K/Akt signaling pathway. Hsp90 inhibition can lead to the degradation of these proteins, resulting in decreased survival signals and increased susceptibility to apoptosis. Several studies have demonstrated that targeting HSP90 can enhance the effectiveness of chemotherapeutic agents like Cisplatin by promoting apoptosis in cancer cells, including HepG2 cells^48,55,56^.

Furthermore, Hsp90 inhibition can stabilize PTEN, a tumor suppressor that negatively regulates the PI3K/Akt pathway. This stabilization of PTEN may contribute to the observed Akt inhibition and enhanced antitumor effects in HepG2 cells. It has been previously reported that HSP90 inhibitors can increase PTEN expression and activity, leading to enhanced apoptosis in various cancer cell lines^57–59^.

It is noteworthy that Hsp90 also maintains the stability and proper folding of GSK-3β, a key regulator of cellular processes. Inhibition of HSP90 can disrupt the interaction between HSP90 and its co-chaperone phosphoglycerate kinase 1 (PGK1), leading to decreased GSK-3β stability and subsequent inhibition^60^. This is significant as GSK-3β inhibition has been shown to contribute to cell death and proliferation in HCC^61–63^.

The differential response between HepG2 and HUH-7 cells indicates a cell line-specific mechanism that warrants further investigation. HepG2 cells may exhibit heightened sensitivity to the combination therapy due to unique genetic and molecular characteristics, such as specific expression profiles or mutations that favor apoptosis through PTEN stabilization. In contrast, HUH-7 cells might possess alternative survival mechanisms or higher baseline levels of anti-apoptotic proteins, which could diminish the effectiveness of the combination therapy. Previous studies have shown varying responses to chemotherapeutic agents among different liver cancer cell lines, highlighting the importance of understanding these differences^64,65^. These findings are visually represented in Fig. 9.

In this study, analysis of TCGA hepatocellular carcinoma data encompassing 424 samples; 365 tumor, 59 normal; has resulted in identification of potential prognostic markers for increased survival of HCC tumor samples. TCGA HCC samples were classified into several groups based on high/low levels of expression of studied molecular markers, with median values of expression levels per sample being used for categorizing HCC tumor samples.

Survival analysis was conducted to assess how HCC patients survive during high/low levels of PTEN, PI3K/AKT/mTOR pathway, β-catenin/BCL2, and β-catenin/HSP90. Results showed that PTEN was a key modulator for PI3K/AKT/mTOR pathway where (PTEN+/PI3K−/AKT−/mTOR−) samples had better overall survival probability compared to (PTEN−/PI3K+/AKT+/mTOR+) samples. This is evident with other findings where reduced PTEN levels were demonstrated to be accompanied by increased PI3K/AKT pathway with increased glucose uptake, tumor cell proliferation, and HCC cell survival^66^.

Also, β-catenin/BCL2 and β-catenin/HSP90 interaction were found to be significantly related to enhanced HCC patients’ survival, where (β-catenin−/BCL2−) samples had better survival chances compared to (β-catenin+/BCL2+). In addition, (β-catenin−/HSP90−) HCC samples had overall significantly higher survival rates compared to (β-catenin+/HSP90+). This is also evident in other studies, where overexpressed IRF2 and downexpressed β-catenin led to decreased BCL2 levels in HCC samples, thus leading to better survival for tumor patients^67^. Also, luminespib (AUY922) treatment led to inhibition of HSP90 and thus reduced accumulation of β-catenin in HCC cells, thus enhanced survival probability for studied patients^68^. Thus, modulation of PI3K/AKT/mTOR pathway through enhanced PTEN, as well as reduced β-catenin/BCL2 and β-catenin/HSP90 expression leads to significantly better survival probability in hepatocellular carcinoma samples.

Finally, the synergistic inhibition of HSP90, PI3K, and Akt pathways in HepG2 cells can lead to enhanced apoptosis. By targeting these pathways, the combination therapy not only reduces cell proliferation but also promotes apoptotic cell death through mechanisms involving modulation of Bcl-2 family proteins, such as Bcl-2. This multifaceted approach reinforces the antitumor effects observed in HepG2 cells, underscoring the potential of this combination therapy in cancer treatment.

This proposed combination of cisplatin and palbociclib (C + P) holds promise for treating hepatocellular carcinoma (HCC) patients with specific molecular characteristics, particularly those with cisplatin-resistant tumors, PTEN loss or PI3K/AKT/mTOR hyperactivation, and RB1-proficient tumors. Notably, this therapeutic strategy may offer a valuable option for intermediate-stage HCC patients who are not candidates for surgery or immunotherapy, as well as those experiencing disease progression following treatment with first-line tyrosine kinase inhibitors (TKIs) such as sorafenib or regorafenib.

## Conclusion

In conclusion, our investigation unveils, for the first time, the anti-cancer potential of combining cisplatin and palbociclib in HCC cells, paving the way for further research on this promising treatment strategy. Our findings demonstrate that the C + P combination treatment significantly outperforms cisplatin alone in suppressing viability, inducing apoptosis, and promoting DNA damage in HCC cells. Mechanistically, the combination treatment may exert its strongest impact in HepG2 cells by synergistically inhibiting HSP90, consequently stabilizing PTEN, and ultimately leading to potent Akt pathway inhibition. PTEN overexpression inhibits PI3K/AKT/mTOR pathway in TCGA HCC samples, thus PTEN acts as a potential modulator for enhancing HCC prognosis. Reduced β-catenin expression along with both BCL2 and HSP90 results in better overall survival for carcinoma patients, thus rendering such molecular markers as prognostic factors for survival of patients following their inhibition using targeted inhibition therapy. The unique response observed in HepG2 cells compared to HUH-7 emphasizes the need for personalized approaches in cancer therapy based on specific cellular mechanisms.

## Electronic supplementary material

Below is the link to the electronic supplementary material.

Supplementary Material 1

Supplementary Material 2

Supplementary Material 3

## Data Availability

All data generated or analysed during this study are included in this published article [and its supplementary information files].
