# Supplementary material for: Cisplatin palbociclib combination differentially modulates PTEN AKT signaling via Hsp90 in hepatocellular carcinoma cells
Source: Sci Rep. 2025 Jun 2;15:19319. doi: 10.1038/s41598-025-04008-1 (PMC12130490; doi:10.1038/s41598-025-04008-1)
Supplement: Supplementary file 1 — Supplementary Material 1 [file 41598_2025_4008_MOESM1_ESM.docx]

| 1. **GAPDH** | 1. **PTEN** |
| --- | --- |
| L 1 2 3 4 5 6 7 8 +ve control c  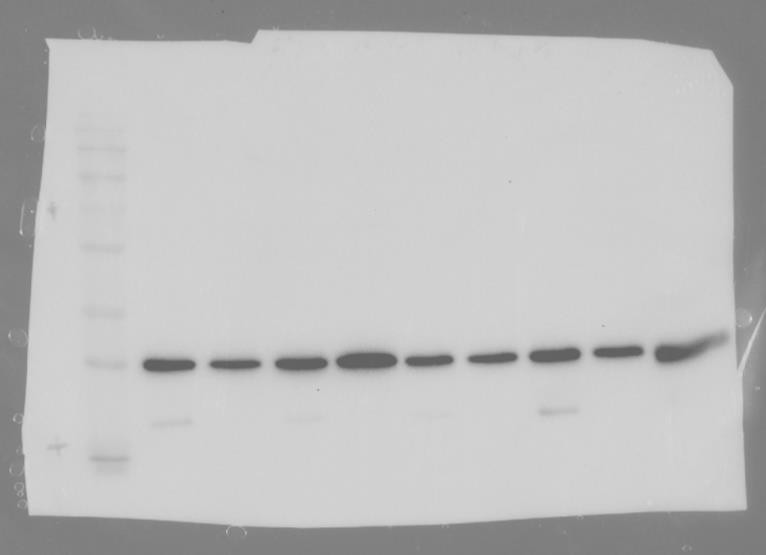 | L 1 2 3 4 5 6 7 8 +ve control  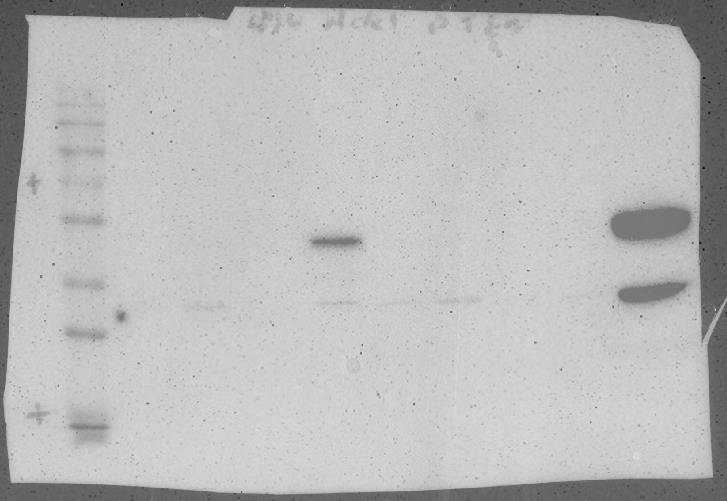 |

| 1. **P-AKT** |
| --- |
| L 1 2 3 4 5 6 7 8 +ve control  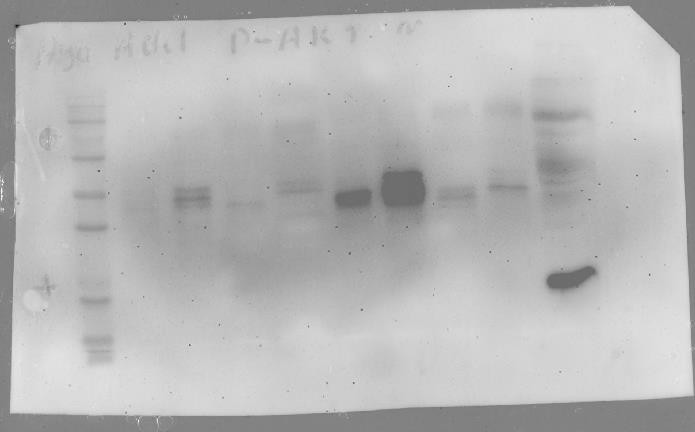 |

**Supplementary Figure [1]: Full membrane images of Western blot analysis of (A) GAPDH, (B) PTEN and (C) p-AKT in HepG2 and HUH-7 cell lines**. Lanes 1-4 represent HepG2 Control, HepG2 Cisplatin, HepG2 Palbociclib, and HepG2 Combination, respectively. Lanes 5-8 represent HUH-7 Control, HUH-7 Cisplatin, HUH-7 Palbociclib, and HUH-7 Combination, respectively. GAPDH was used as a loading control. These images are uncropped and represent the entire membrane area used for analysis.
