# Supplementary material for: Cisplatin palbociclib combination differentially modulates PTEN AKT signaling via Hsp90 in hepatocellular carcinoma cells
Source: Sci Rep. 2025 Jun 2;15:19319. doi: 10.1038/s41598-025-04008-1 (PMC12130490; doi:10.1038/s41598-025-04008-1)

**Supplementary Figure 2. Survival analysis for high/low molecular markers in TCGA HCC samples.**

(2.1) KM Plot for TCGA HCC samples (β-catenin+/BCL2- vs β-catenin-/BCL2+). (2.2) KM Plot for TCGA HCC samples (β-catenin+/HSP90- vs β-catenin-/HSP90+).

**2.1**

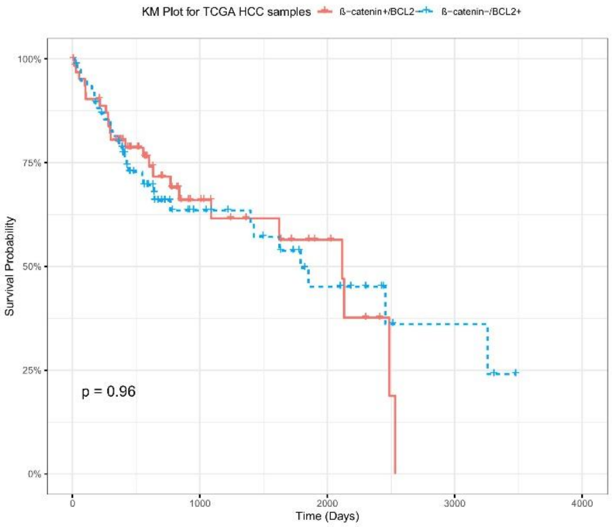


**2.2**

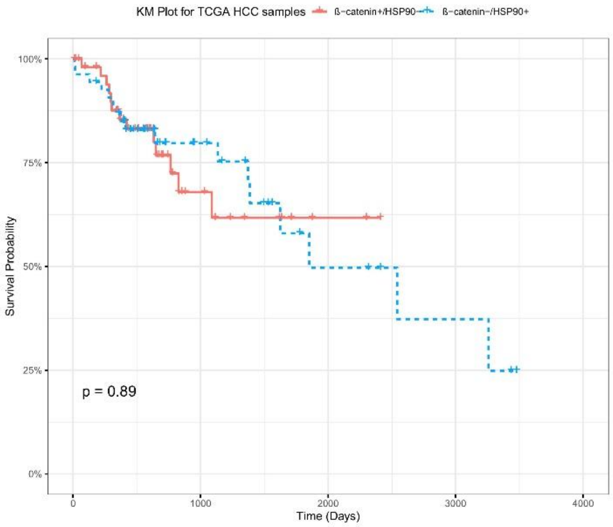

Supplement: Supplementary file 2 — Supplementary Material 2 [file 41598_2025_4008_MOESM2_ESM.pdf]
